# Efficacy of 8-week oral iron supplementation on fatigue and physical capacity in young women with iron deficiency anemia: An uncontrolled pilot clinical trial

**DOI:** 10.1371/journal.pone.0334499

**Published:** 2025-10-16

**Authors:** Mohamed Achraf Harrabi, Thouraya Fendri, Fatma Chaari, Rahma Ayed, Atyh Hadadi, Wissal Boughattas, Ines Mezghani, Choumous Kallel, Haithem Rebai, Mouna Turki, Fatma Ayadi, Sonia Sahli

**Affiliations:** 1 Research Laboratory Education, Motricité, Sport et Santé, EM2S, LR19JS01, High Institute of Sport and Physical Education of Sfax, University of Sfax 3000, Tunisia; 2 Sport, Physical Activity, Rehabilitation and Movement for Performance and Health (SAPRéM), Université d’Orléans, Orléans, France; 3 Complexity, Innovations, Motor and Sports Activities (CIAMS), Université d’Orléans, Orléans, France; 4 Laboratory ‘Movement, Interactions, Performance’, Faculty of Sciences and Technologies, Le Mans University, Le Mans, France; 5 Research Laboratory, Molecular Bases of Human Pathology, LR19ES13, Faculty of Medicine of Sfax, University of Sfax, Sfax, Tunisia; 6 Laboratory of Biochemistry, Habib Bourguiba University Hospital, University of Sfax 3000, Sfax, Tunisia; 7 Sport sciences Department, College of Education, Taif university, Taif, Saudi Arabia; 8 Sport Heath Department, College of Sport Sciences and Physical Activity, Princess Nourah bint Abdulrahman University, Riyadh, Saudi Arabia; 9 Laboratory of Biochemistry, Hedi Chaker University Hospital, University of Sfax 3000, Sfax, Tunisia; 10 Laboratory of Hematology, Habib Bourguiba University Hospital, University of Sfax, Sfax, Tunisia; 11 Sports Performance Optimization (LR09SEP01), National Center of Medecine and Science in Sports (CNMSS), Tunis, Tunisia; University of Rijeka Faculty of Medicine: Sveuciliste u Rijeci Medicinski fakultet, CROATIA

## Abstract

This study aims to investigate the effect of 8 weeks of ferrous sulfate supplementation (160 mg elemental iron/day) on fatigue and physical performance in young women with iron deficiency anemia (IDA). In this uncontrolled pilot clinical trial, twenty-three women with IDA aged between 18 and 30 years participated in this study. Aerobic fitness, muscle strength and muscle endurance were performed to evaluate physical capacities. Moreover, the multidimensional fatigue inventory (MFI-20) was used to assess general, physical, and mental fatigue, reduced activity and motivation. Due to multiple comparisons, the level of significance was set at 0.00625. The results of this study revealed that muscle endurance values increased significantly (P < 0.001) after 8 weeks of iron supplementation compared to pre-intervention values. In addition, scores of general (P < 0.001), physical (P < 0.001) and mental (P < 0.001) fatigue, and reduced activity (P < 0.001) and motivation (P = 0.006) decreased significantly in post- intervention compared to pre- intervention. However, there were no improvements in aerobic fitness (P = 0.008) and muscle strength (P = 0.086). In women of reproductive age with IDA, 8 weeks of iron supplementation improve muscle endurance, but not aerobic fitness or muscle strength. These improvements could be explained by the increase in hemoglobin (Hb) (by 17.62%) and serum ferritin (by 63.2%) concentrations and the decrease in fatigue scores after the supplementation period.

## Introduction

Iron deficiency anemia (IDA) is a decrease in red blood cell production linked to low iron stores in the body. It is the most common nutritional disorder worldwide, accounting for around half of all anemia cases [1,2]. Women of reproductive age are highly vulnerable to IDA, not only due to iron loss in menstrual blood, but also probably because their dietary iron intake may often be low as they may follow restrictive dietary practices to lose weight [3]. The most reported symptom of IDA is fatigue [4] resulting from reduced muscle function [5] and endurance capacity [3]. In this sense, iron deficiency itself can have detrimental effects on muscle function and oxidative energy metabolism [6], leading to reduced exercise and endurance capacity [7]. Moreover, poor muscle oxygenation due to anemia can have deleterious consequences on physical health [8]. IDA treatments include increased intake of iron to replete iron stores, but increasing amounts of food-based iron may not be sufficient given iron content in foods and issues with absorption [9]. Oral iron supplementation, the first-line treatment for iron deficiency, is effective and inexpensive when taken appropriately at 100–200 mg elemental iron per day in 2–3 divided doses [10,11]. A wide range of iron compounds and preparations are available. Ferrous sulfate is an effective and inexpensive treatment [1].

Some studies investigated the effect of iron supplementation on physical capacity (mainly aerobic fitness) in women of reproductive age [12,13]. However, most of these studies have been carried out on iron-deficient women without anemia. Only very few trials were carried out regarding the effect of iron supplementation on muscle performance (*i.e.,* muscle endurance and strength) in other populations (*e.g.,* elderly with IDA) [6].

Physical performance (*e.g.,* endurance, aerobic capacity, or fatigue) has been related to societal outcomes (*e.g.,* social participation, childcare etc.), daily life activities and work performance in patients with IDA [14]. Therefore, exploring the effect of iron supplementation on fatigue and physical capacities (*i.e.,* aerobic fitness, muscle endurance, and strength) in young women would be essential to reduce fatigue, enhance work productivity, social life and daily life activities. The aim of this study was to investigate the effect of 8 weeks of oral iron supplementation on fatigue and physical performance in young women with IDA. We hypothesized that oral iron supplementation with ferrous sulfate for 8 weeks would reduce fatigue and improve physical capacity in young women with IDA.

## Methods

### Participants and study design

Twenty-three women (in apparent good health) with IDA, aged between 18 and 30 years, were recruited in the present study. Recruitment strategy included oral and written advertisements (e.g., flyers) in the Leather and Footwear Vocational Training Center of Sfax. To control any bias related to daily activities, dietary habits/intake and sleep-wake rhythms, all women selected to participate in this study were recruited from the Students’ Residence of this vocational training center. The eligibility criteria were absence of declared chronic disease; chronic medical treatment; blood donation in the last 4 months; pregnancy or breastfeeding; smoking; vitamin, mineral or herbal supplements in the 3 months prior to the beginning of the study and throughout its duration. Women with inflammation (C-reactive protein (CRP) > 5 mg/L) or suffering from severe anemia (hemoglobin (Hb) < 80 g/L) were not included in the study.

This study ran from October 26 to December 22, 2023. For ethical reasons (as it is unacceptable to leave patients suffering from confirmed IDA without appropriate treatment), this study was an uncontrolled clinical trial. After being informed about the experimental procedures, risks and benefits, all women signed an informed written consent form. This study was approved by the local ethics committee (Committee of persons protection of the south, approval number: CPP SUD N° 0213/2020) and it was conducted in accordance with the ethical standards of the Helsinki Declarations. The design of this study also integrated the evaluation of postural control when it was approved by the ethics committee. However, to align more closely with the objectives of this research, postural control was excluded from the current investigation. The study was prospectively registered at the Pan African Clinical Trial Registry (pactr.samrc.ac.za) with study number PACTR202310850205072, on 03/10/2023.

The study protocol consisted of 2 test sessions: the first one 24 h before the intervention (pre-intervention) and the second 24 h after 8 weeks of intervention (post-intervention). During both test sessions, analyses of complete blood count (CBC), CRP and serum ferritin were performed. In addition, lower limb muscle strength and endurance, aerobic fitness and fatigue were assessed by the same experimenters. The principal investigator and the physician involved in our research team make weekly visits to monitor compliance with iron supplementation by counting supplement tablets, and to record and monitor side effects of ferrous sulfate.

### Intervention program

Each woman was asked to take 494.5 mg ferrous sulfate per day, as oral supplementation, corresponding to 160 mg elemental iron per day [11,15]. The supplement was administered in divided doses (247.25 mg ferrous sulfate 2 times a day) over an 8-week period. Participants were requested to avoid eating one hour before and after iron supplementation in order to optimize iron absorption. The first dose was administered in the morning on an empty stomach, with breakfast taken 1 h after supplementation. The second dose was taken at least 1 h after the last food intake and before going to sleep. To ensure that the results obtained were exclusively due to the effects of iron supplementation, without any influence from physical or athletic activities that might distort the findings of this study, participants were asked not to do any supplemental physical or sports activities during the intervention.

### Laboratory analyses

Blood samples were collected for all participants in the morning between 8 and 9 am after at least 12 hours of fasting. These samples were obtained by venipuncture of the antecubital vein and analyzed in the biochemistry laboratory of CHU Habib Bourguiba in Sfax on the same day. CBC was measured using a Sysmex® XN-1000 hematology analyzer. Serum ferritin assessment (reagent reference: 03737551190) was performed on a Roche Cobas® e 601. CRP (reagent reference: 04956842190) was assessed on the Roche Cobas® c 501 to detect the presence of inflammation (CRP > 5 mg/L). Anemia was diagnosed according to WHO criteria, which require an Hb concentration < 12 g/dL for women. Anemic women with ferritin concentrations below 15 μg/L were diagnosed with IDA.

### Fatigue assessment

Fatigue was assessed using the French version of the Multidimensional Fatigue Inventory (MFI) [16]. The MFI is a valid, reliable, and highly feasible self-questionnaire for women with anemia [17]. It consists of 20 questions designed to assess fatigue in several dimensions. It includes 5 items (4 questions each) covering general fatigue, physical fatigue, mental fatigue, reduced activity, and motivation. The score for each item is obtained by the summation of the scores relating to the questions in the corresponding item. Higher scores indicate higher levels of fatigue.

### Muscle endurance assessment

The Killy test assesses static lower limbs muscle endurance, particularly the thighs [18]. First, a description and demonstration of the required position were presented, followed by a familiarization test. Beforehand, the participant positioned herself with her back against the wall, and the examiner checked the angle of the right knee (90° flexion) with a goniometer and marked the position of the right heel on the floor with a marker. The participant then rested for about 30 s to eliminate the fatigue accumulated during the position test. She was then invited to reposition herself, taking into account the mark on the floor. After a quick check of the knee angle, the examiner started the stopwatch while the participant maintained this position as long as possible. The criterion for stopping the test was the loss of thigh horizontality. A first warning about the position is allowed. The recorded performance corresponded to the time taken to hold this position in seconds.

### Aerobic fitness assessment

Aerobic fitness was assessed using the shuttle test. This test consists of running back and forth until exhaustion between two lines placed 20 m apart. Beeps were used to increase the running speed by 0.5 km.h^-1^ every minute from a starting speed of 8.5 km.h^-1^. The test stopped when the participant was no longer able to keep up with the set pace. The last level announced (*i.e.,* the equivalent maximum aerobic speed) is then used as the VO2max indicator. VO2max (mL.kg.min^-1^) was estimated from the table given by Léger et al. for adult men and women aged over 18 [19].

### Isometric muscle strength assessment

The quadriceps maximal force (in Kg) of the dominant leg was measured using an isometric force-measuring chair adapted for the lower limb. The participant was seated on the chair with knees and hips flexed to 90°, and the trunk, thigh and pelvis secured by straps to minimize uncontrolled movement (lateral, vertical or frontal). A padded ankle strap, attached to a connected electronic traction dynamometer (K-Force^®^ Link, KINVENT, Montpellier, France), was adjusted to 2 cm above the lateral malleolus of the dominant leg. Once correctly positioned, participants were asked to perform 3 maximal voluntary isometric contractions of the quadriceps (extension of the leg in relation to the thigh) after a light warm-up of a few contractions. Each repetition was held for 5 s with a 2 min rest between trials. The best force value of the 3 trials was adopted as the representative value of the maximum voluntary isometric contraction (MVIC). Verbal encouragement was provided throughout the trials.

### Sample size calculation

Given the lack of published data in the literature, an estimate of sample size was calculated using a large effect size (Cohen’s d = 0.8), corresponding to a substantial within-subject difference relative to the expected variability. This estimate was chosen based on conventional thresholds for a large effect and reflects our expectation of a clinically meaningful change in pre- and post-intervention differences in fatigue and physical capacity. Using G * power software (version 3.1.9.2; University of Kiel, Kiel, Germany), it was estimated that a minimum of 21 participants would be needed (using the statistical t-test: means; difference between two dependent means) considering an alpha of 0.00625 (p-value adjusted to control for running multiple interactions of the primary outcomes) and a power of 0.8.

### Statistical analyses

Statistical analyses were conducted using IBM SPSS^®^ statistics software version 25. To verify normality of variances the Shapiro-wilk test was performed. For variables with normal distribution, data were presented as mean ± standard deviation, and parametric analyses (paired sample T-test) were performed. For variables with non-normal distribution, data were presented as median (interquartile range) and non-parametric analyses (Wilcoxon test) were used. Effect sizes were calculated using the eta-square (η^2^) for the Wilcoxon test and the Cohen’s d for the paired sample T-test [20]. The level of significance was set at P < 0.05. However, In order to control running multiple interactions of the primary outcomes, adjustment of the p-value about 0.00625 (p-value of 0.05 divided by eight univariate tests) was applied.

## Results

**Fig 1:**
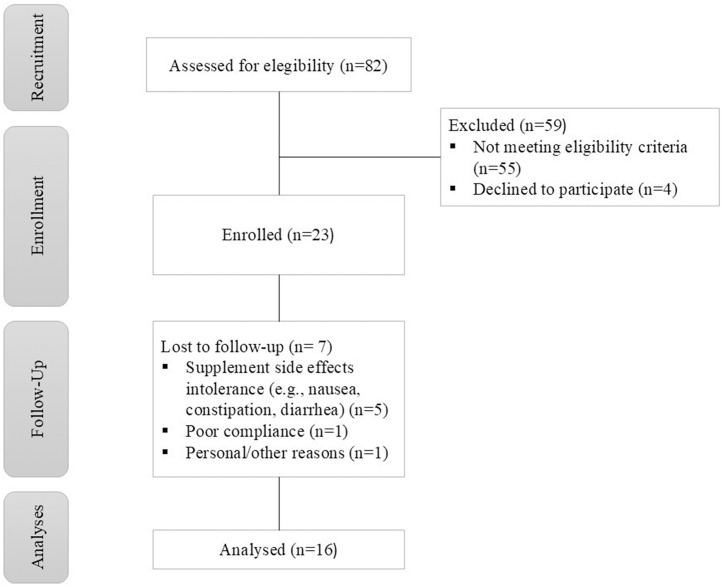
Flow chart of study participants.

Twenty-three women with IDA, aged between 18 and 30 years, were recruited in the present study. During the intervention, there were 7 withdrawals and data of the sixteen participants left (age: 21.69 ± 1.92 years, weight: 55.78 ± 10.5 kg, height: 1.61 ± 0.07 m, body mass index: 21.32 ± 2.88) were included in statistical analysis. A flow diagram showing participant recruitment and reasons for withdrawals is in Fig 1.

In women with IDA who took 494.5 mg ferrous sulfate daily for eight weeks, Hb concentration [mean difference (MD) = 2.18 g/dL (95% CI: 1.67, 2.68), P < 0.001], serum ferritin concentration [MD = 2.19 μg/L (95% CI: 12.64, 21.98), P < 0.001], and muscle endurance [MD = 3.09 s (95% CI: 3.39, 16.57), P = 0.006], increased over time. Conversely, scores for general fatigue [MD = −4.69 (95% CI: – 5.8, −3.57), P < 0.001] physical fatigue [MD = −4.88 (95% CI: −5.88, −3.87), P < 0.001], mental fatigue [P < 0.001], reduced activity [MD = −3.63 (95% CI: −4.61, −2.63), P < 0.001] and reduced motivation [P = 0.006] decreased over time. However, no significant difference was found for quadriceps muscle strength values [MD = −0.03 kg (95% CI: −0.39; 0.33), P = 0.86] nor for aerobic fitness [P = 0.008] before and after 8 weeks of iron supplementation (Table 1).

**Table 1:** Data of hemoglobin (Hb) and serum Ferritin (sF) concentrations, muscle endurance, maximal voluntary isometric contractions (MVIC), aerobic fitness (VO2max) and fatigue (MFI) in women with iron deficiency anemia (IDA) pre- (Pre) and post-supplementation (Post).

	Pre (n = 16)	Post (n = 16)	Δ%	t/z Value	P value	Effect size d/ η^2^
Hb (g/dL)	10.19 (1.27)	12.37 (0.97)	17.62%	t = −9.26	P < 0.001	d = 1.93 ^c^
sF (μg/L)	8.87 (3.3)	26.19 (9.19)	63.2%	t = −7.9	P < 0.001	d = −2.33 ^c^
Muscle endurance (s)	26.43 (14.95)	36.41 (12.13)	28.34%	t = −3.23	P < 0.006	d = −0.72 ^b^
MVIC (kg)	24.77 (6.65)	24.74 (6.64)	−0.25%	t = 0.18	P = 0.86	d = 0.005 ^a^
VO2max (mL.kg.min^-1^)	26.6 (26.6, 29.6)	29.6 (27.35, 29.6)	4.32%	z = −2.65	P = 0.008	η^2^ = 0.44 ^c^
MFI (score 4–20)						
General fatigue	15 (1.82)	10.31 (1.35)	−47.5%	t = 8.98	P < 0.001	d = 2.9 ^c^
Physical fatigue	15.56 (1.67)	10.68 (1.62)	−48.18%	t = 10.3	P < 0.001	d = 2.96 ^c^
Mental fatigue	15 (13.25, 16)	10.12 (1.26)	−45%	z = −3.54	P < 0.001	η^2^ = 0.78 ^c^
Reduced activity	13.62 (1.86)	10 (1.03)	−37.19%	t = 7.81	P < 0.001	d = 2.34 ^c^
Reduced motivation	13.5 (13, 14.75)	10.5 (9.25, 12)	−29.21%	z = −2.72	P < 0.006	η^2^ = 0.46 ^c^

Data are presented as mean (SD) or median (Q1, Q3).

t value, statistical value of T-test; z value, statistical value of Wilcoxon test; d, Cohen’s d effect size value for parametric test (T-test); η^2^, eta square value effect size value for non-parametric test (Wilcoxon test).

^a^small effect, ^b^medium effect, ^c^large effect.

## Discussion

The aim of this study was to explore the effect of 8 weeks of iron supplementation on fatigue and physical capacities in women with IDA. The results of the current study revealed significant improvements in muscle endurance and all fatigue items in post- compared to pre-intervention. Nevertheless, no improvement in quadriceps muscle strength nor in aerobic fitness were observed after 8 weeks of iron supplementation. Furthermore, the results of the present study demonstrated significant increases in Hb and serum ferritin concentrations (S1 Table).

Iron is a crucial trace element required for oxygen delivery to tissues and oxygen utilization at cellular and subcellular levels. It is a functional component of iron-containing proteins, especially Hb, myoglobin, cytochromes, and specific iron-containing enzymes. Thus, the improvement in Hb concentrations could explain the significant decreases in general fatigue and reduced activity scores after 8 weeks of iron supplementation. Furthermore, iron plays an essential role in energy utilization [21]. In this context, iron depletion is known to impair physical aerobic performance through a reduction in maximal oxygen consumption (VO2max) by the body during intense exercise [22]. The decrease in VO2max in patients with anemia is mainly due to a low Hb concentration and the consequent reduction in the oxygen-carrying capacity of the blood [22]. Our results revealed a tendency to increase VO2max by 4.32%. However, this increase is not significant. These finding are in discordance with those of Radjen et al. who found improvements in VO2max [23] by 18.2% following 8 weeks of 200 mg ferrous sulfate supplementation. The supplementary effect of physical training on aerobic capacity reported in Radjen et al. study may explain this difference. Thus, it would be of interest to consider combining physical activity with iron supplementation to have a better enhancement of aerobic capacity.

Concerning muscle performance, it has been demonstrated that the iron-depleted cells have reduced activities of citrate synthase, aconitase, isocitrate dehydrogenase (IDH) and succinate dehydrogenase (SDH), leading to a decrease in nicotinamide adenine dinucleotide (NADH) production [24]. These cells have an impaired capacity to generate adenosine triphosphate (ATP) by oxidative phosphorylation, which is subsequently expressed by reduced oxygen consumption of mitochondria during iron-deficient conditions. In contrast, iron-replete cells display an optimized Krebs cycle and stimulate mitochondrial respiration, leading to increased oxygen consumption and ATP synthesis [24]. In this sense, iron depletion leads to a reduction in NADH formation and, consequently, ATP production [24]. To overcome this lack of oxidative ATP synthesis, cells must intensify glycolysis to form NADH and ATP by anaerobic mechanisms [25]. This allows the production of pyruvate, that is then enzymatically converted to lactate and NAD^+^, which could explain muscle fatigue observed in women with IDA [26]. Iron supplementation enhances Hb concentrations and consequently oxygen transport capacity, decreasing subsequently the excessive conversion of pyruvate to lactate due to anaerobiosis reduction. Therefore, blood lactate concentrations decrease pre- and post-exercise [27]. In fact, this phenomenon may help understand the reduction in physical fatigue recorded in women with IDA in our study in post- compared to pre-supplementation and, consequently, the enhancement of muscle endurance. However, the results of the present study showed no significant improvement in quadriceps muscle strength after the supplementation period. This result could be due to the typology and specificity of the muscle fibers. In fact, it’s known that type I and IIA fibers are rich in mitochondria, and primarily use the cell’s oxidative capacity to promote greater resistance to fatigue. On the other hand, type II fibers have a lower density of mitochondria and more glycolytic than oxidative capacity, providing high muscle strength but low fatigue resistance [28]. In this sense, it seems that the possible improvement in muscular oxidative capacity has mostly stimulated type I and IIA muscle fibers, which could explain the improvement in endurance but not muscle strength.

It was reported that women with IDA had higher mental fatigue scores than controls [26,29]. These studies stated that mental fatigue is mainly due to reduced cognitive capacity as a result of poor cerebral oxygenation and reduced iron bioavailability caused by IDA [30]. Hence, it can be suggested that the improvement in iron bioavailability after 8 weeks of iron supplementation, as well as the increase in Hb concentrations, found in our study, may be at the origin of the reduction in mental fatigue scores. In addition, it seems that all the aforementioned improvements contributed to a better psychological state and improved motivation in our patients after iron supplementation.

Our results emphasize the importance of iron supplementation to reduce fatigue and enhance physical capacity through enhancing VO2max and muscle endurance. These improvements are crucial to improve daily life activities, work performance, social life and consequently life quality in women with IDA. However, no improvement in muscle strength was observed, suggesting the potential need for physical activity to enhance efficacy. Future research should explore the impact of combining iron supplementation with physical activity on muscle performance in women with IDA.

This study faces some limitations. A control group of women with IDA receiving a placebo during the intervention period would have enabled us to accurately control the effects of supplementation on the various parameters measured. The main causes of this limitation are ethical considerations and medicolegal obligations. Furthermore, the reader should interpret the results and particularly the p-values with caution as this is, in fact, a small pilot trial. The nature of this study is due to the fact that we solicited all women from the same students’ residence of the vocational training center, for all participants recruitment. The choice of recruiting the young women of this same residence center was made to avoid all potential bias, related to daily activities, dietary habits/intake and sleep-wake rhythms. This latter explains the reason why this study’s sample size was relatively small, as we couldn’t recruit more participants from other centers. Hence, the sample size is limited, and this restricts the generalizability of the findings. Further studies with larger, more representative samples are needed to validate these preliminary findings and to better understand the mechanisms by which supplementation may improve physical and muscular capacities in women with IDA.

## Conclusion

This uncontrolled pilot study revealed that 8 weeks of fractional iron supplementation (247.25 mg ferrous sulfate 2x/day) improved aerobic fitness and muscle endurance in young women with IDA, but not muscle strength. These physical improvements could be explained by increased Hb and serum ferritin concentrations, as well as lower fatigue scores.

## Supporting information

S1 Table Participants data. (XLSX)

S1 File Trends checklist. (DOCX)

S2 File Protocol trial. (DOCX)
