# Supplementary material for: Efficacy of 8-week oral iron supplementation on fatigue and physical capacity in young women with iron deficiency anemia: An uncontrolled pilot clinical trial
Source: PLoS One. 2025 Oct 16;20(10):e0334499. doi: 10.1371/journal.pone.0334499 (PMC12530587; doi:10.1371/journal.pone.0334499)
Supplement: S2 File — (DOCX) [file pone.0334499.s003.docx]

**Project summary**

Iron deficiency anemia (IDA) is the most prevalent nutritional disorder in the world [1, 2]. Due to iron loss during menstruation, women of reproductive age are highly vulnerable to IDA [3]. This hematological disorder has been associated with decreased physical performance, including endurance, and increased fatigue, which can negatively affect postural control, social interactions and childcare responsibilities [4]. Therefore, exploring the effects of iron supplementation on postural control, fatigue and physical performance (*i.e.,* aerobic fitness, muscle endurance, and strength) in young women is essential for reducing fatigue and enhancing work productivity, social interactions, and daily activities. Thus, this study’s aim is to evaluate the effects of an 8-week oral iron supplementation (494.5 mg ferrous sulfate on two divided doses) on postural control, fatigue and physical performance in young women with IDA. This study’s time frame is from October to December 2023. Sixteen women with IDA, aged between 18 and 30 years, will participate in this study. The center of pressure oscillations and aerobic fitness, muscle strength and endurance will be performed to evaluate postural control and physical capacities respectively. Moreover, the multidimensional fatigue inventory (MFI) will be used to assess general, physical, and mental fatigue, reduced activity and motivation. Eight weeks of iron supplementation would improve aerobic fitness, muscle performance, postural control, decrease fatigue, and increase hemoglobin (Hb) and serum ferritin levels in women with IDA.

**General information**

- Protocol title: Postural control, iron deficiency anemia and physical activity in adult women: exploration and treatment
- Name and address of the sponsor/funder: None.
- Name and title of the investigator who is responsible for conducting the research, and the address and telephone number of the research site, including responsibilities of each.
  - Dr. Mohamed Achraf Harrabi
  - Research Laboratory Education, Motricité, Sport et Santé, EM2S: High Institute of Sport and Physical Education of Sfax, airport road, 3.5 km, 3000, Sfax, Tunisia; telephone number: +216 74 505 702.
- Names and addresses of the clinical laboratories and other medical and/or technical departments and/or institutions involved in the research.
  - Research Laboratory Education, Motricité, Sport et Santé, EM2S, LR19JS01, High Institute of Sport and Physical Education of Sfax, University of Sfax 3000, Tunisia.
  - Laboratory 'Movement, Interactions, Performance', Faculty of Sciences and Technologies, Le Mans University, Le Mans, France.
  - Research Laboratory, Molecular Bases of Human Pathology, LR19ES13, Faculty of Medicine of Sfax, University of Sfax, Sfax, Tunisia
  - Laboratory of Biochemistry, Habib Bourguiba University Hospital, University of Sfax 3000, Sfax, Tunisia.
  - Sport sciences department, college of education, Taif university, Taif, Saudi Arabia.
  - Laboratory of Biochemistry, Hedi Chaker University Hospital, University of Sfax 3000, Sfax, Tunisia.
  - Laboratory of Hematology, Habib Bourguiba University Hospital, University of Sfax, Sfax, Tunisia.
  - Sports Performance Optimization (LR09SEP01), National Center of Medecine and Science in Sports (CNMSS), Tunis, Tunisia.
- **Rationale & background information**

Some investigations explored the effect of iron supplementation on physical capacity (primarily aerobic fitness) in women of reproductive age [5, 6]. However, the majority of these studies have been conducted on iron-deficient women without anemia. Little evidence exists concerning the effect of iron supplementation on muscle performance (*i.e.,* muscle endurance and strength) in other populations (*e.g.,* elderly with IDA) [7]. Moreover, physical performance (*e.g.,* endurance, aerobic capacity, or fatigue) and postural control has an impact on social life (*e.g.,* social participation, childcare etc.), daily life activities and work performance in individuals with IDA [4]. Therefore, investigating the effect of iron supplementation on fatigue, physical capacities (*i.e.,* aerobic fitness, muscle endurance, and strength) and postural control in young women with IDA would be fundamental to reduce fatigue, enhance work productivity, social life and daily life activities.

**Study goals and objectives**

This study aims to investigate the effect of 8 weeks of oral iron supplementation (160 mg of elemental iron) on fatigue, physical performance and postural control in young women with IDA.

**Study design**

This study was an uncontrolled clinical trial conducted on young women with iron-deficiency anemia, aged between 18 and 30 years, with no comorbidities. The study’s expected duration is 3 months.

**Methodology**

The study protocol consisted of 2 measurements sessions: the first one 24 h before the intervention (pre-intervention) and the second one 24 h after 8 weeks of intervention (post-intervention) (Figure 1). During both test sessions, lower limb muscle strength (K-Force dynamometer) and endurance (Killy test), aerobic fitness (Leger 20-meter shuttle run test), postural control (Stabilotest force platform: TechnoConcept®) and fatigue (Multidimensional Fatigue inventory) were evaluated by the same experimenters. Additionally, analyses of complete blood count (CBC), C-reactive protein (CRP) and serum ferritin were conducted.

Women will be requested to take 494.5 mg ferrous sulfate per day, as oral supplementation, corresponding to 160 mg elemental iron per day [8, 9]. The supplement will be administered in divided doses (247.25 mg ferrous sulfate 2 times a day) for 8 weeks. Patients will be asked to avoid eating 1 h before and after iron supplementation to optimize iron absorption. The first dose will be administered in the morning on an empty stomach, with breakfast taken 1 h after supplementation. The second dose will be taken at least 1 h after the last food intake and before sleeping. Participants won’t do any additional physical or sports activities during the intervention.


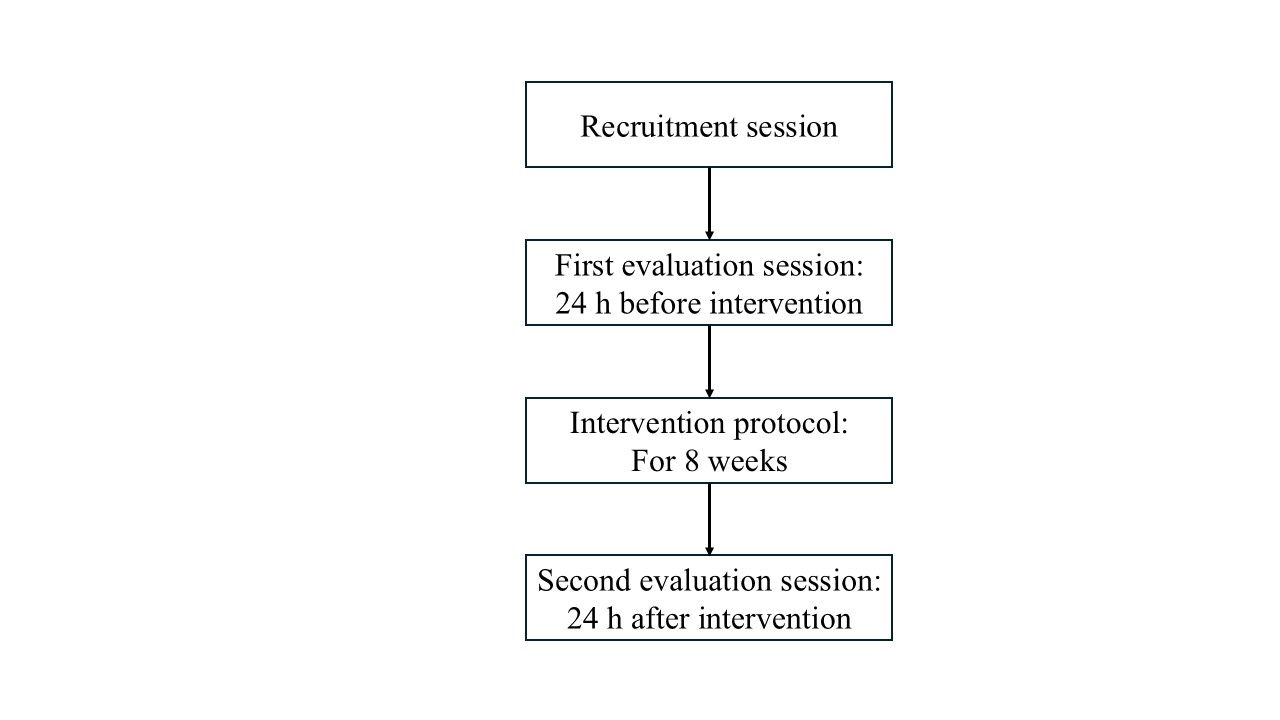


**Figure 1.** Flow diagram

**Safety considerations**

Starting from recruitment, a medical doctor followed all participants, recorded and reported all events until the end of the study.

**Follow-up**

Participants were followed up daily, until the end of the second session measurements by phone calls or by visiting them directly. The supplementation intake was checked by counting the pills left in the tablet every week.

**Data management and statistical analysis**

An a priori estimate of sample size calculation using an effect size of 0.8 indicated that at least 15 participants are needed to detect differences in pre- and post-intervention measures for women with iron deficiency anemia (IDA). This was determined using G*Power software (version 3.1.9.2) with an alpha level of 0.05 and a power of 0.9. All the data for this study were collected by the examiners and recorded on a computerized excel spreadsheet. After this step, all data were checked by the principal investigator to ascertain that there were no errors. Statistical analyses were performed using IBM SPSS®, version 25. The Shapiro-Wilk test will assess normality. For normally distributed variables, results will be presented as mean ± standard deviation, and paired sample T-tests will be conducted. For non-normally distributed variables, data will be expressed as median (interquartile range), and Wilcoxon test will be used. Effect sizes will be calculated via eta-squared (η²) for the Wilcoxon test and Cohen's d for the T-test. Significance is set at P < 0.05.

**Quality assurance**

A clinical monitor will oversee the progress of the clinical trial, and ensure that it is conducted, recorded, and reported in accordance with the protocol and good clinical practice (GCP).

**Expected outcomes of the study**

Women with IDA would have improved aerobic fitness and muscle performance, decreased fatigue, enhanced postural control and increased hemoglobin (Hb) and serum ferritin levels in women after 8 weeks of iron supplementation. The results of this study will provide clinicians with recommendations about reducing fatigue and enhancing physical capacity and postural control in women with IDA.

**Duration of the project**

October 2 to 24: recruitment phase

October 25: first evaluation session

October 26 to December 21: experimental phase (iron supplementation)

December 22: second evaluation session

**Problems anticipated**

To avoid any bias linked to daily activities, eating habits and sleep-wake rhythms, all the women will be recruited from the same vocational training center and live in the same accommodation center.

**Project management**

Mohamed Achraf Harrabi, Sonia Sahli, Fatma Ayadi, Haithem Rebai and Mouna Turki conceived and designed the experiments. Mohamed Achraf Harrabi, Thouraya Fendri, Fatma Chaari and Rahma Ayed will perform the experiments. Rahma Ayed, Ines Mezghanni and Choumous Kallel will recruit patients and collect blood samples. Rahma Ayed, Fatma Chaari, Choumous Kallel and Ines Mezghanni will analyze the data. Sonia Sahli, Atyh Hadadi and Wissal Boughattas will critically review the manuscript. All authors will read and approve the final manuscript.

**Ethics**

This study will be conducted in accordance with the ethical standards of the Helsinki Declarations. After being informed about the experimental procedures, risks and benefits, all participants will sign an informed written consent form (Appendix).

**References**

1. Warner, M.J. and M.T. Kamran, *Iron Deficiency Anemia*, in *StatPearls*. 2022, StatPearls Publishing, Copyright © 2022, StatPearls Publishing LLC.: Treasure Island (FL).

2. WHO and Unicef, *Iron deficiency anaemia: assessment, prevention control, a guide for programme managers*. 2001, World Health Organization: Geneva. p. 1-114.

3. Coad, J. and K. Pedley, *Iron deficiency and iron deficiency anemia in women.* Scandinavian Journal of Clinical and Laboratory Investigation, 2014. **74**(sup244): p. 82-89.

4. Haas, J.D. and T.t. Brownlie, *Iron deficiency and reduced work capacity: a critical review of the research to determine a causal relationship.* J Nutr, 2001. **131**(2s-2): p. 676S-688S; discussion 688S-690S.

5. Michael Sze Yuan Low, et al., *Daily iron supplementation for improving anaemia, iron status and health in menstruating women.* Cochrane Database Syst Rev., 2016.

6. Pasricha, S.R., et al., *Iron supplementation benefits physical performance in women of reproductive age: a systematic review and meta-analysis.* J Nutr, 2014. **144**(6): p. 906-14.

7. Neidlein, S., R. Wirth, and M. Pourhassan, *Iron deficiency, fatigue and muscle strength and function in older hospitalized patients.* Eur J Clin Nutr, 2021. **75**(3): p. 456-463.

8. Pasricha, S.R., et al., *Diagnosis and management of iron deficiency anaemia: a clinical update.* Med J Aust, 2010. **193**(9): p. 525-32.

9. Pavord, S., et al., *UK guidelines on the management of iron deficiency in pregnancy.* Br J Haematol, 2012. **156**(5): p. 588-600.

**Research protocol: part 2**

**Budget**

The main investigator of this study funded the supplementation.

**Collaboration with other scientists or research institutions**

None

**Links to other projects**

None

**Other research activities of the investigators**

None

**Appendix**

FORMULAIRE DE CONSENTEMENT DESTINE AUX PARTICIPANTS AU PROTOCOLE DE RECHERCHE SCIENTIFIQUE

**Titre de l’étude** : *Contrôle postural, anémie ferriprive et activité physique chez les femmes adultes : Exploration et traitement.*

Je soussigné(e) :

**Nom** :……………………………………………………**Prénom**………………………………………………………………

**Adresse** : …….…………………......................................................................………………………………….

**Numéro de téléphone** :……………………………………………………

Atteste par le présent consentement que :

- J’accepte librement et volontairement de participer à cette étude scientifique.
- J’ai pris connaissance du protocole de cette recherche optionnelle et de l’intérêt de récupérer des prélèvements sanguins dans le cadre de cette étude scientifique.
- J’ai pu poser toutes les questions que je voulais, j’ai reçu des réponses adaptées et j’ai pu disposer d’un temps de réflexion suffisant entre l’information et la décision de ma participation à cette étude.
- Si je me retire volontairement de l’étude, le chercheur pourra utiliser les informations et les échantillons recueillis jusque-là, sauf opposition de ma part formulée au moment de ce retrait.
- Les résultats de cette recherche pourraient être publiés dans des revues scientifiques ou lors des congrès scientifiques.

Je déclare avoir **lu** et **compris** les termes de la présente formule

Fait à Sfax, le……/.…/………

**Signature**

CONSENT FORM FOR PARTICIPANTS IN THE SCIENTIFIC RESEARCH PROTOCOL

**Title of study:** Postural control, iron deficiency anemia and physical activity in adult women: Exploration and treatment.

I, the undersigned:

**First name** :…………………………………………………… **Last name:**…………………………………………………

**Adress** : …….…………………......................................................................………………………………….

**Phone number** :……………………………………………………

Hereby certify that :

- I freely and voluntarily agree to participate in this scientific study.
- I have been informed of the protocol for this optional research and of the benefits of collecting blood samples for this scientific study.
- I was able to ask all the questions I wanted, received appropriate answers and had sufficient time to reflect between the information and the decision to take part in this study.
- If I voluntarily withdraw from the study, the researcher will be able to use the information and samples collected up to that point, unless I express my opposition at the time of my withdrawal.
- The results of this research may be published in scientific journals or at scientific conferences.

I declare that I have **read** and **understood** the terms of this form

Signed in Sfax, on ……/……/…..……

**Signature**
